# CD300a Regulates Mouse Macrophage Functionality in Allergic Inflammation

**DOI:** 10.1159/000529606

**Published:** 2023-03-16

**Authors:** Pier Giorgio Puzzovio, Bruce D. Levy, Francesca Levi-Schaffer

**Affiliations:** a Pharmacology and Experimental Therapeutics Unit, School of Pharmacy, Institute for Drug Research, Faculty of Medicine, The Hebrew University of Jerusalem, Jerusalem, Israel; b Pulmonary and Critical Care Medicine, Department of Internal Medicine, Brigham and Women’s Hospital, Harvard Medical School, Boston, MA, USA

**Keywords:** Allergic inflammation, CD300a, Macrophages, Mast cells

## Abstract

**Background:**

CD300a is an inhibitory receptor (IR) expressed on several leukocytes, including mast cells (MCs) and macrophages (MΦ), important cells in allergic inflammation (AI). We have previously characterized CD300a role on MCs and in vivo in mouse models of allergy, in which the absence of CD300a resulted in increased inflammatory features and delayed resolution. However, the exact mechanism of this delayed resolution is unclear. Our hypothesis is that MΦ, important players in resolution, might be impaired when CD300a is absent.

**Objectives:**

The aim of the study was to investigate CD300a-dependent functionality of mouse MΦ.

**Method:**

MΦ were purified from the peritoneum of wild-type (WT) and CD300a^−/−^ mice naïve and 48 h and 96 h after challenge with ovalbumin/alum. Phenotype switching was analyzed via specific M1-M2 inducers and markers. MΦ phagocytotic ability was assessed via *Staphylococcus aureus* pHrodo-conjugated bioparticles. The influence of MCs on MΦ was investigated by incubating WT MΦ with supernatants from non-activated and IgE-activated bone marrow-derived MCs (BMMCs) and analyzing functional responses.

**Results:**

Naïve CD300a^−/−^ MΦ presented with increased sensitivity to activation when treated with LPS. Absence of CD300a results in increased Arg1 expression and increased IL-6 release when MΦ are purified from allergic peritonitis-induced mice. Similar results were obtained when CD300a^−/−^ MΦ were purified 96 h after challenge. On the other hand, CD300a absence did not affect phagocytosis. WT MΦ incubated with supernatants of non-activated and IgE-activated BMMCs presented with increased iNOS expression and decreased Arg1 levels.

**Conclusions:**

The IR CD300a controls the activation state of MΦ, and its absence could augment the inflammatory state seen in CD300a^−/−^ mice. Moreover, MCs can also influence MΦ phenotype switching. This may partially explain the delayed AI resolution seen in these mice.

## Introduction

CD300a is an inhibitory receptor belonging to the CD300 family of surface glycoproteins. It is expressed on most immune cells and notably on mast cells (MCs) and eosinophils (Eos) [1], cardinal effector cells of allergic inflammation (AI). In our previous works, we have extensively characterized its functionality in AI. Activation of CD300a on MCs and Eos exerts anti-inflammatory effects by inhibiting cell functionality [2]. In mouse models of acute and chronic airway AI, passive cutaneous anaphylaxis, and stem cell factor-induced peritonitis models, administration of bispecific anti-CD300a/anti-IgE, anti-CD300a/anti-CCR3, or anti-CD300a/anti-cKit anti-bodies, synthesized to specifically activate this IR on MCs or on Eos, was shown to inhibit several markers of AI [3–5]. Moreover, and most importantly, we have more recently shown that CD300a^−/−^ mice with allergic peritonitis (AP) show increased cell infiltration and delayed resolution, mainly characterized by higher numbers of Eos in the peritoneal cavity [6]. Resolution of inflammation is defined as the period between the infiltration of inflammatory cells at the site of damage and their clearance [7]. Acute AI is self-limited by the process of its resolution. In case of chronic AI, resolution mechanisms fail and inflammation prevails, as seen in several kinds of allergic diseases such as asthma and atopic dermatitis (AD) [8].

We hypothesized that lack of CD300a might affect the pro-resolution properties of cells such as the macrophages (MΦ), important cells both in inflammation and in its resolution. In resolution, MΦ can phagocytize dead inflammatory cells, secrete anti-inflammatory mediators, and switch phenotype from a pro-inflammatory M1 toward a pro-resolutory M2 [9–11]. Notably, human [12] and mouse [13] MΦ have been shown to express CD300a. In human MΦ, CD300a was discovered to have a negative effect on the uptake of dead cells [12], while on C57BL mice peritoneal MΦ it did not mediate phagocytosis [14].

In the present work, we sought to clarify the reasons for delayed resolution in AP in CD300a^−/−^ mice. We investigated this by characterizing wild-type (WT) and CD300a^−/−^ MΦ obtained from CD300a^−/−^ mice peritoneal lavage for phenotype and activation features in basal/ naïve conditions and during OVA/alum-induced AP.

## Materials and Methods

### Mice

C;129S5 *Cd300a*^*tm1Lex*^/Mmucd mice (referred to as CD300a^−/−^) were generated at MMRC UC Davis MMRC, University of California, Davis, CA, originally donated by Genentech. The CD300a gene consists of 7 exons. Coding exons 2 and 3 were targeted by homologous recombination. Mice backcrossed starting at N4 to BALB/c mice for 10 generations. WT BALB/c mice were obtained from Harlan Laboratories (Rehovot, Israel) and grown in-house. In all experiments, gender (female) and age-matched mice (7–8-week old) were used and housed under specific pathogen-free conditions. All mouse experiments were approved by the Animal Experimentation Ethics Committee of the Hebrew University of Jerusalem and performed in accordance with the guidelines of the committee.

### Bone Marrow-Derived MCs

Bone marrow-derived MCs (BMMCs) were obtained from bone marrows of 7–8-week-old female BALB/c mice. In brief, BM was obtained from the femurs of the mice after dissection in sterile conditions. Dispersed cells were cultured for 4 weeks to obtain mature BMMCs. Prior to experimentation, BMMCs were assessed for viability (by trypan blue exclusion) and for 90% maturity by acidic toluidine blue staining and expression of characteristic surface markers (cKit and FcεRI) by flow cytometry. Cells were used at >95% viability and >90% maturity.

BMMCs were sensitized overnight with mouse IgE anti-dinitrophenyl (Sigma-Aldrich, Rehovot, Israel) (0.5 μg/mL). BMMCs were then washed and resuspended in Tyrode’s buffer containing Ca^2+^ and Mg^2+^ (TB^2+^). Cells (1–2*10^5^/well) were plated in 96 U-shaped plates (Corning), incubated at 37°C, and activated with dinitrophenyl-BSA (50 ng/mL) for 1 h. After activation, cells and supernatants were separated by centrifugation 1,250 rpm, 5 min, 4°C.

### Materials

All plasticwares were purchased from Thermo Fisher Scientific (MA, USA).

### Mouse OVA/Alum AP

WT and CD300a^−/−^ BALB/c (7–9-week-old) female mice were anesthetized by isoflurane inhalation and subcutaneously sensitized with 100 mg of OVA (Sigma-Aldrich) and 1.6 mg of alum (Sigma-Aldrich) in 200 μL of PBS on days −14 and −7. On day 0, mice were challenged intraperitoneally (i.p.) with 200 μL of PBS containing 10 mg of OVA alone. Control mice received PBS i.p. Mice were euthanized by CO_2_ inhalation, 48 h and 96 h days after challenge. The peritoneal cavity was washed with 3 mL of cold PBS + 3% fetal calf serum. The total volume injected was considered for the evaluation of cell population numbers. Lavages were centrifuged (5 min, 4°C, 250 g), and supernatants were collected and stored (−80°C) to assess cytokine levels. Cells were employed for MΦ purification. The results of this model are shown in [6].

### Peritoneal MΦ Purification and Phenotyping

Peritoneal lavages from WT and CD300a^−/−^ (BALB/c, females, 7–9 weeks old) OVA-challenged mice euthanized 48 or 96 h after challenge were collected from 6 different mice and pooled and seeded in 6-well plates. In some experiments, peritoneal MΦ were purified from naïve WT and CD300a^−/−^ mice. Lavages were incubated at 37°C for 3 h to let cells adhere. After incubation, cells were washed 3 times with warm PBS to remove non-adherent cells. Adherent cells (MΦ) were cultured o.n. or for 24 h with RPMI containing 10% (vol/vol) fetal calf/bovine serum, penicillin (100 U ml^−1^), streptomycin (100 μg mL^−1^). For co-culture experiments, 1*10^5^ MΦ were seeded in 96-U-shaped-wells plates and cultured 24 h with supernatants from either not activated or IgE-activated BMMCs. Where indicated, LPS (1 μg/mL) (Sigma-Aldrich, Rehovot, Israel) or recombinant mouse IL-4 (10 ng/mL) (PeproTech Asia, Israel) was added to the culture medium. After incubation, cells were harvested with a cell scraper and counted in a hemacytometer. Cell suspensions were centrifuged, and supernatants were collected to assess cytokine release. Cell pellets were stored at −80° for RNA extraction.

### RNA Extraction and RT-PCR

Total RNA was extracted from WT and CD300a^−/−^ cell pellets via Quick-RNA Miniprep Kit (ZymoResearch, CA, USA) according to manufacturer’s instructions. RNA concentration was assessed via Nanodrop ND-1000 (Thermo Fisher Scientific, MA, USA). cDNA was prepared from total RNA via qScript cDNA Synthesis Kit (Quantabio, MA, USA) following manufacturer’s protocol. RT-PCR was performed on cDNA from WT and CD300a^−/−^ MΦ with specific primers for the M1/M2 marker genes and mouse Arg1 (Fw: 5ʹ-AGCACTGAGGAAAGCTGGTC-3ʹ; Rv: 5ʹ-TACGTCTCGCAAGCCAATGT-3ʹ) (Sigma-Aldrich, Rehovot, Israel). Actin was used as a housekeeping gene for internal reference (Fw: 5′-TCAC-CAACTGGGACGCATG; 5′-GTACAGGGATAGCACAGCCT). Gel pictures were acquired via Bio-Rad ChemiDoc XRS (Bio-Rad, CA, USA) and analyzed via Image Lab software (Bio-Rad).

### Cytokine Release

IL-10 and IL-6 release from supernatants and peritoneal lavages of WT and CD300a^−/−^ MΦ were assessed with mouse IL-10 Standard ABTS ELISA Development Kit (detection range: 47–3,000 pg/mL; PeproTech Asia), mouse IL-6 Standard ABTS ELISA Development Kit (detection range: 62–4,000 pg/mL; PeproTech Asia), and mouse IL-4 Standard ABTS ELISA Development Kit (detection range: 31–2,000 pg/mL; PeproTech Asia) according to manufacturer’s instructions.

### Phagocytosis Assay

Phagocytotic ability of WT and CD300a^−/−^ steady-state peritoneal MΦ was assessed by employing pHrodo^™^ Red *S. aureus* BioParticles^®^ Conjugate (Thermo Fisher Scientific) according to manufacturer’s instructions. Control samples were kept on ice to prevent phagocytosis. Cells were acquired with FACSCalibur (Becton Dickinson, NJ, USA) through the FL2 channel and analyzed with FlowJo software.

### Statistical Analysis

Data are expressed as mean ± SEM. Statistical comparisons were performed using Student “*t*” test or unpaired “*t*” test. For more than three experimental groups, ANOVA followed by post hoc Tukey test was performed. Data were analyzed with Microsoft Excel (Microsoft, Washington, USA). A two-tailed “*p*” value of less than 0.05 was considered statistically significant for all analyses. GraphPad QuickCalc outlier calculator software was employed in order to detect outliers.

## Results

### Peritoneal Naive MΦ Phenotyping in WT and CD300a^−/−^ Mice

**Fig. 1.**
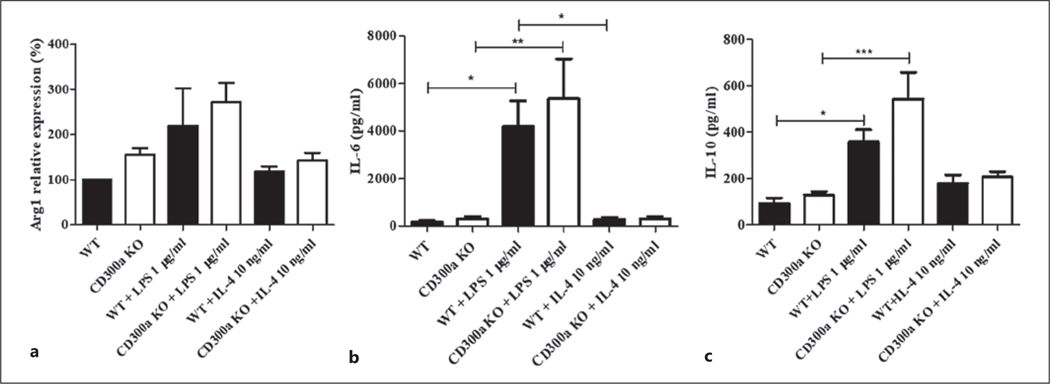
Peritoneal naive MΦ phenotyping in WT and CD300a^−/−^ mice. **a** Arginase 1 expression in macrophages purified from the peritoneal cavity of WT and CD300a^−/−^ (CD300a KO) mice. IL-6 (**b**) and IL-10 (**c**) levels in the supernatants of cultured WT and CD300a^−/−^ macrophages not activated and activated with either LPS (1 μg/mL) or IL-4 (10 ng/mL). Data are expressed as mean ± SEM of three independent experiments (*n* = 2 mice/group/ experiment; *p < 0.05, **p < 0.01, ***p < 0.001).

In order to investigate the reason for the delayed resolution in CD300a^−/−^ mice, we attempted to characterize naive WT and CD300a^−/−^ MΦ phenotype. We found that CD300a^−/−^ MΦ expressed slightly more Arg1 than WT MΦ (shown in Fig. 1a) and released significantly more IL-6 and slightly but not significantly more IL-10 than WT MΦ (shown in Fig. 1b, c). Interestingly, Arg1 expression, and IL-6 and IL-10 release were increased in CD300a^−/−^ in comparison with WT MΦ after activation with LPS (shown in Fig. 1b, c).

Treatment with IL-4 did not significantly change Arg1 expression or IL-6 release (shown in Fig. 1a, b), but it significantly increased IL-10 release from CD300a^−/−^ MΦ (shown in Fig. 1c). These results might indicate that the lack of CD300a enhances MΦ responses to inflammatory stimuli.

### Peritoneal MΦ Phenotyping in an OVA/Alum AP Model in WT and CD300a^−/−^ Mice 48 and 96 h after OVA Challenge

**Fig. 2.**
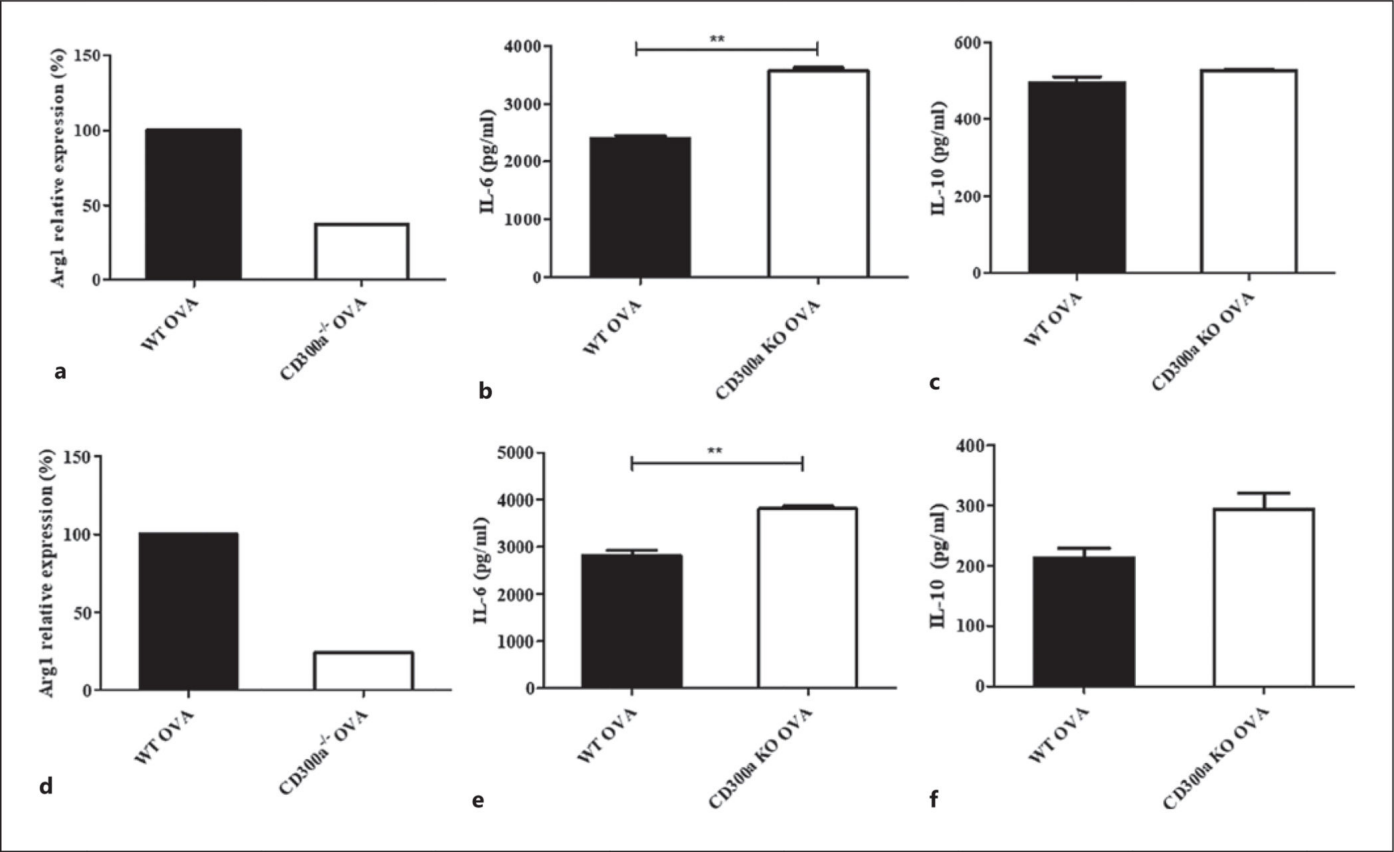
Peritoneal MΦ phenotyping in an OVA/alum AP model in WT and CD300a^−/−^ mice 48 h and 96 h after OVA challenge. **A** Arginase 1 expression in macrophages purified from the peritoneal cavity of WT and CD300a^−/−^ (CD300a KO) mice 48 h after challenge. IL-6 (**b**) and IL-10 (**c**) levels in the supernatants of cultured WT and CD300a^−/−^ macrophages purified 48 h after challenge. **d** Arginase 1 expression in macrophages purified from the peritoneal cavity of WT and CD300a^−/−^ mice 96 h after challenge. IL-6 (**e**) and IL-10 (**f**) levels in the supernatants of cultured WT and CD300a^−/−^ macrophages purified 96 h after challenge. Data are expressed as mean ± SEM of two independent experiments (*n* = 5–6 mice/group; **p < 0.01).

In order to explain the delayed resolution of AI we previously reported [6], we investigated the phenotype of WT and CD300a^−/−^ MΦ in the context of AI. We performed a well-established OVA/alum AP model, in which we detected a peak of inflammation at 96 h post-challenge, as shown by total cell and Eos numbers in the peritoneal cavity. These results are reported in [6]. CD300a^−/−^ MΦ purified 48 h after challenge from OVA-challenged mice showed lower expression of Arg1 (80% reduction) in comparison with WT MΦ (shown in Fig. 2a). IL-6 release was slightly but significantly increased in CD300a^−/−^ comparison with WT MΦ, while IL-10 release did not significantly change (shown in Fig. 2a–c). Activation with LPS significantly increased both IL-6 and IL-10 release from CD300a^−/−^ MΦ in comparison with WT MΦ (shown in online suppl. Fig. 1a, b; for all online suppl. material, see www.karger.com/doi/10.1159/000529606). IL-6 and IL-10 production was significantly increased in CD300a^−/−^ than in WT MΦ after IL-4 treatment (shown in online suppl. Fig. 1a, b). We detected similar features when MΦ were harvested from WT and CD300a^−/−^ mice 96 h after challenge. CD300a^−/−^ MΦ presented with reduced Arg1 expression (shown in Fig. 2d) and increased IL-6 (shown in Fig. 2e) and IL-10 (shown in Fig. 2f) release, with IL-6 being higher in concentrations than IL-10. These data point out that CD300a^−/−^ MΦ, together with their increased sensitivity to activation, are less prone to switch to the M2 phenotype in the context of AI.

### Phagocytosis Is Not Affected by Lack of CD300a

**Fig. 3.**
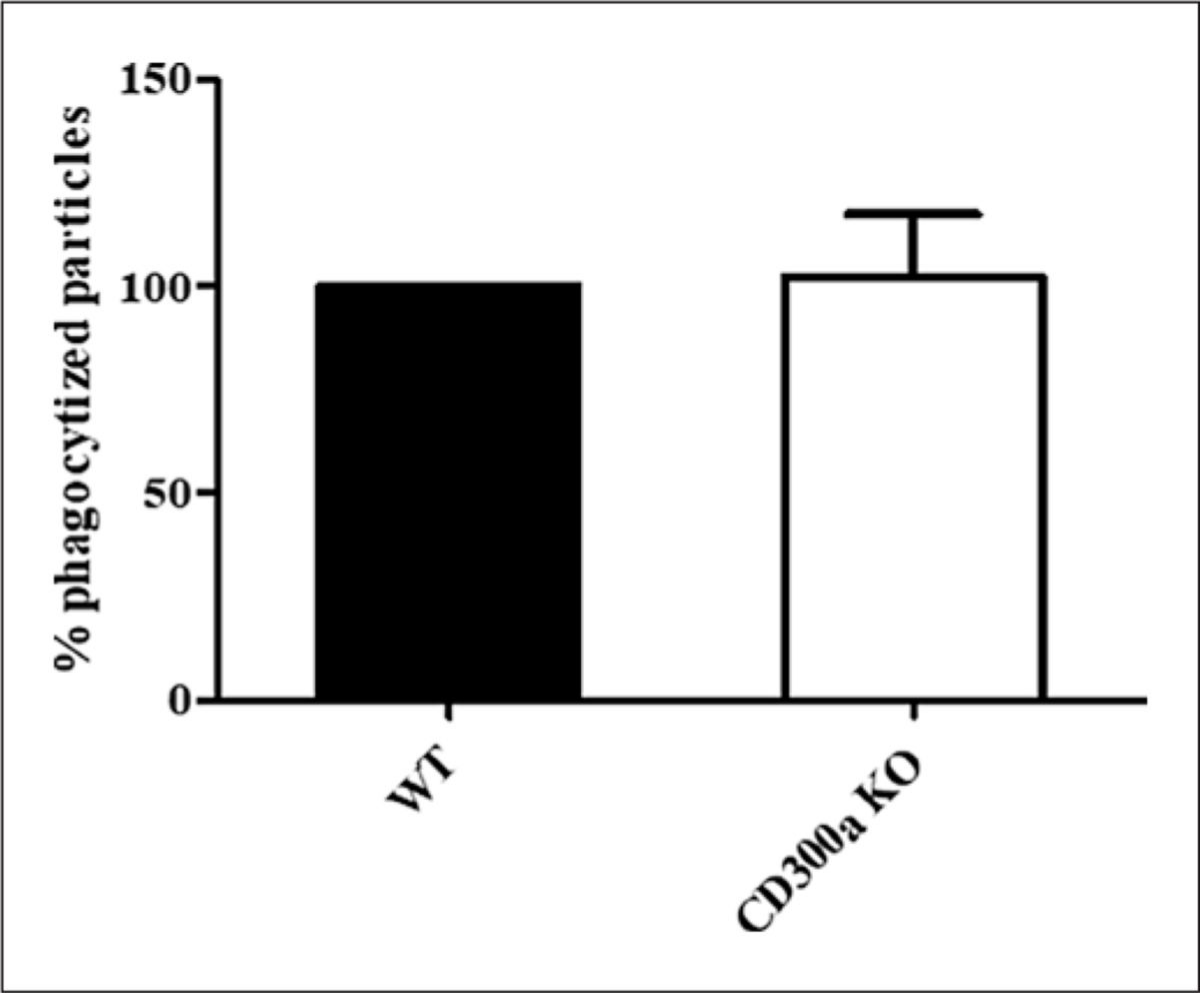
Phagocytosis is not affected by lack of CD300a. Percentage of *S. aureus* bioparticles phagocytized by peritoneal MΦ from WT and CD300a^−/−^ (CD300a KO) mice. Data are expressed as mean ± SEM of three independent experiments.

In view of the reduced possibility of CD300a^−/−^ MΦ to switch to an M2 phenotype, we hypothesized that these MΦ might have impaired phagocytosis. We then performed a phagocytosis assay using *S. aureus* bioparticles conjugated to a pH-sensitive fluorescent dye. We did not detect any differences in phagocytic ability between WT and CD300a^−/−^ MΦ (shown in Fig. 3). This might indicate that lack of CD300a affects the activation features of MΦ without impairing their main function.

### Co-Culture of Not Activated and Activated BMMC Supernatants with WT MΦ

Next, in order to understand how the interaction with other immune cells might affect MΦ phenotype switching in the context of AI, we incubated WT MΦ with supernatants from IgE-activated and non-activated BMMCs. We aimed to culture the MΦ in the presence of the mediators released by MCs in order to mimic the microenvironment occurring during AP. Our findings show that WT MΦ present with increased iNOS expression when incubated with IgE-activated BMMC supernatants rather than non-activated BMMC ones (shown in online suppl. Fig. 2a). On the other hand, incubation with BMMC supernatants abolished Arg1 expression (shown in online suppl. Fig. 2b). This might indicate that the effect of the interaction with other immune cells during AI might selectively modulate some features of MΦ phenotype.

## Discussion

In our previous works, we characterized the expression of CD300a in human AD skin and in mouse models of AP and AD. Given its function as an IR on MCs and on Eos, we found that the absence of CD300a resulted in increased inflammation and delayed in vivo resolution of these AI responses [6, 15]. However, the mechanism underlying this delayed resolution is still unclear. One possibility is that MΦ, the main cells involved in resolution of inflammation, might be functionally impaired when CD300a is knocked out. Indeed, when CD300a^−/−^ BMMCs incubated with apoptotic bone marrow-derived Eos, their release of pro-inflammatory mediators is not inhibited (Cohen-Mor S., Shamri R., Levi-Schaffer F., unpublished data). Therefore, in this work we aimed to characterize CD300a^−/−^ MΦ in the context of AI.

Our first question was whether naïve WT and CD300a^−/−^ are functionally different. Therefore, we purified peritoneal MΦ from CD300a^−/−^ mice and induced phenotype switching by incubation with either LPS or IL-4 to test their functional properties. Our results show that naïve CD300a^−/−^ MΦ display a slight increase, albeit not significant, in Arg1 expression in comparison with WT MΦ. Interestingly, CD300a^−/−^ MΦ showed significantly increased IL-6 release, while IL-10 release was augmented only when CD300a^−/−^ MΦ were treated with either LPS or IL-4. Similar results were obtained when naïve CD300a^−/−^ MΦ were activated with the specific M1 or M2 inducer, as lack of CD300a induced increased IL-6 and IL-10 release from activated MΦ. These results reconfirm our previous report showing that MΦ lacking CD300a display increased IL-6 release after LPS-mediated activation [16]. In the same paper, however, IL-10 release from splenocytes was found to be decreased in the absence of CD300a [16]. Nakahashi-Oda et al., on the other hand, demonstrated that IL-10 release was augmented after CD300a^−/−^ MΦ from C57BL mice were incubated with apoptotic cells and activated with LPS [14]. Next, we characterized CD300a^−/−^ MΦ in the context of AI by purifying MΦ from the peritoneal cavity of CD300a^−/−^ AP mice 48 h after OVA challenge and assessing their phenotype. The OVA/alum-induced AP is a model we have extensively performed in our laboratory. As published in [6], this model consists of two OVA/alum sensitizations separated by 1 week and one OVA challenge. The peak of inflammation occurs 96 h after the challenge, and its main hallmarks are the increase in total peritoneal cell numbers and Eos infiltration, a typical feature of AI. Noteworthy, MCs and MΦ numbers do not change significantly during the course of the model, and this prompted us to hypothesize that both MCs and MΦ can switch their functionality from pro-inflammatory to pro-resolutory and possibly influence each other in this model of AI. Interestingly, expression of Arg1 was greatly decreased on these CD300a^−/−^ MΦ while IL-6 release was significantly increased, and IL-10 release was not affected. Since Arg1 is indicative of the pro-resolution M2 phenotype, this result might hint that when CD300a is absent, MΦ are less able to switch to the M2 phenotype during an AI event. Similar results were obtained from CD300a MΦ purified 96 h after challenge and after incubation with LPS or IL-4. This is the first time that CD300a^−/−^ MΦ have been shown to be less able to switch to the M2 phenotype in AI. As for the modulation of IL-6 and IL-10 release in all the examined conditions, it is reasonable to infer that lack of CD300a might affect MΦ functionality as previously reported for mouse BMMCs, by increasing the release of pro-inflammatory and anti-inflammatory mediators [6]. In addition, we previously reported that engagement of CD300a with an activating antibody results in increased IL-10 release [17]. From all this evidence, we can infer that CD300a on MΦ might regulate their ability to switch phenotype when AI is induced. Moreover, lack of CD300a results in enhanced sensitivity to activating stimuli.

Next, we analyzed phagocytotic ability of MΦ when CD300a is absent. Surprisingly, no differences were detected between WT and CD300a^−/−^ MΦ in phagocyting *S. aureus* bioparticles, which express phosphatidylserine, the putative ligand for mouse CD300a. This is not surprising, since also in a previous report no differences were found between WT and CD300a^−/−^ ability to phagocytize apoptotic cells in C57BL mice [14]. However, in previous reports, CD300a^−/−^ mice presented with impaired phagocytosis of fluorescent beads by neutrophils [18] and defective efferocytosis of apoptotic neutrophils by peritoneal MΦ [19]. It is possible that CD300a differentially influences phagocytosis and efferocytosis on different cells according to the target employed (beads or apoptotic cells).

Our next step was to understand whether MΦ phenotype switching might be influenced by other immune cells. We chose MCs as they are the main initiator and effector cells that persist in AI development [20]. Therefore, we cultured the MΦ in the presence of the mediators released by MCs to mimic the microenvironment occurring during AP. Our results show that when WT MΦ are incubated with supernatants from non-activated and IgE-activated BMMCs, iNOS expression increases while Arg1 expression is almost abolished. This is in disagreement with a previous work in which MC-derived proteases were found to induce M2 phenotype on MΦ [21]. Another recent report showed that exogenous histamine enhances expression and production of CCL18, a chemokine with both pro- and anti-inflammatory effects in AI [22], from M2 human MΦ [23]. Nevertheless, the different data might be due to MC supernatants being collected 1 h after their activation. In this short time frame, MCs secrete a plethora of pre-formed pro-inflammatory mediators. At later time points, MCs release different mediators that might differentially influence the phenotype of MΦ, by inducing a switch from M1 to M2 phenotype during the course of AI.

In conclusion, we have brought forth evidence of a novel function of CD300a that might regulate MΦ activity by influencing their phenotype switching. This might account for the delayed resolution of inflammation previously described by us and others, possibly bolstering the fact that CD300a could be immunopharmacologically targeted to modulate the function of the cells involved in AI.

## Supplementary Material

Supplementary Material text

Figure S1

Figure S2

## Data Availability

All data generated or analyzed during this study are included in this article and its online supplementary material files. Further inquiries can be directed to the corresponding author.
