# Supplementary material for: CD300a Regulates Mouse Macrophage Functionality in Allergic Inflammation
Source: Int Arch Allergy Immunol. Author manuscript; Available in PMC 2024 Mar 16. (PMC10350930; doi:10.1159/000529606)
Supplement: Supplementary Material text [file NIHMS1898991-supplement-Supplementary_Material_text.docx]

**CD300a regulates mouse macrophages functionality in allergic inflammation**

**Pier Giorgio Puzzovio^1^, Bruce D. Levy^2^, Francesca Levi-Schaffer^1^*.**

^1^ Pharmacology and Experimental Therapeutics Unit, School of Pharmacy, Institute for Drug Research, Faculty of Medicine, The Hebrew University of Jerusalem, Jerusalem, Israel.

^2^ Pulmonary and Critical Care Medicine, Department of Internal Medicine, Brigham and Women's Hospital, Harvard Medical School, Boston, Massachusetts, USA

*Corresponding author: Prof. Francesca Levi-Schaffer, Pharmacology and Experimental Therapeutics Unit, Institute for Drug Research, School of Pharmacy, Faculty of Medicine, The Hebrew University of Jerusalem, P.O.B. 12272, Jerusalem 91120, Israel, E-mail: francescal@ekmd.huji.ac.il, Tel: +972-2-6757512, Fax: +972-2-6758144

**SUPPLEMENTAL FIGURE LEGENDS**

**Supplemental Fig. 1: Peritoneal CD300a-/- MΦ purified from OVA/Alum AP model present with increased mediators’ release.**

(**a**) IL-6 and (**b**) IL-10 levels in the supernatants of cultured WT and CD300a^-/-^ macrophages purified 48 h after challenge. MΦ were not activated or activated with either LPS (1 µg/ml) or IL-4 (10 ng/ml). Data are expressed as mean ± SEM of two independent experiments (n=5-6 mice/group; *p<0.05, **p<0.01, ***p<0.001).

**Supplemental Fig. S2: Co-culture of not activated and activated BMMC supernatants with WT MΦ affects MΦ phenotype switching.**

iNOS (**a**) and Arg1 (**b**) expression in peritoneal WT MΦ after 24h incubation with medium alone, supernatants from not activated (NA) and DNP-BSA-activated (AC) BMMCs (representative of n=2). Data is expressed as mean ± SEM.
