# Supplementary figures and images for: CD300a Regulates Mouse Macrophage Functionality in Allergic Inflammation
Source: Int Arch Allergy Immunol. Author manuscript; Available in PMC 2024 Mar 16. (PMC10350930; doi:10.1159/000529606)

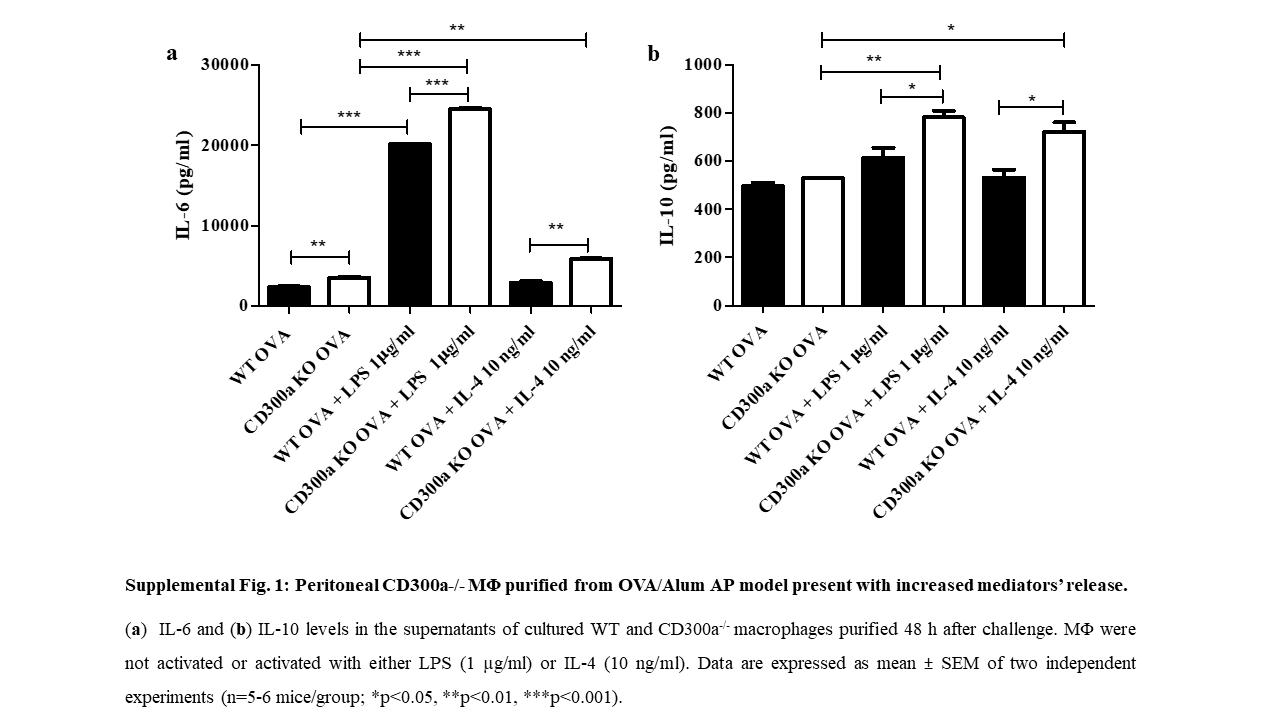

Supplement: Figure S1 [file NIHMS1898991-supplement-Figure_S1.tif]

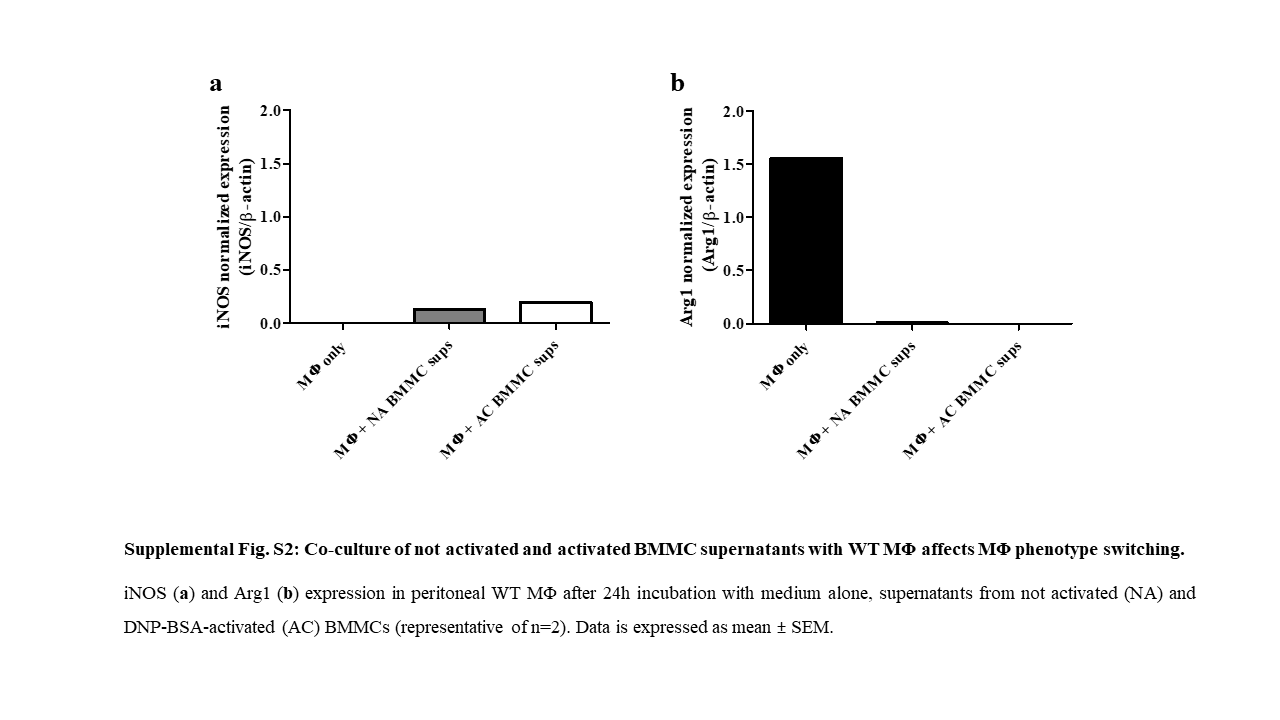

Supplement: Figure S2 [file NIHMS1898991-supplement-Figure_S2.tif]
